# Epilepsy surgery for children with epileptic spasms: A systematic review and meta‐analysis with focus on predictors and outcomes

**DOI:** 10.1002/epi4.13007

**Published:** 2024-07-02

**Authors:** Taylor Kolosky, Andrea Goldstein Shipper, Kai Sun, Busra Tozduman, Soren Bentzen, Ahsan N. Moosa, Gozde Erdemir

**Affiliations:** ^1^ University of Maryland School of Medicine Baltimore Maryland USA; ^2^ Cooper Medical School of Rowan University Camden New Jersey USA; ^3^ Institute for Clinical and Translational Research University of Maryland Baltimore Baltimore Maryland USA; ^4^ Department of Public Health Dokuz Eylul University Izmir Turkey; ^5^ Department of Neurology The Charles Shor Epilepsy Center, Neurological Institute, Cleveland Clinic Cleveland Ohio USA; ^6^ Division of Pediatric Neurology and Department of Neurology University of Maryland Medical Center, University of Maryland School of Medicine Baltimore Maryland USA

**Keywords:** epileptic spasms, infantile spasms, pediatric, prognostic factors, resective surgery, seizure freedom, West syndrome

## Abstract

To conduct a systematic review of the literature regarding rates and predictors of favorable seizure outcome after resective surgery for epileptic spasms (ES) in pediatric patients. The Preferred Reporting Items for Systematic Reviews and Meta‐Analyses standards were followed. We searched PubMed, EMBASE, and Cochrane CENTRAL for articles published on the prevalence or incidence of epileptic spasm since 1985. Abstract, full‐text review, and data extraction were conducted by two independent reviewers. Meta‐analysis was performed to assess overall seizure freedom rate. Subject‐level analysis was performed on a subset of studies to identify prognostic indicators. A total of 21 retrospective studies (*n* = 531) were included. Meta‐analysis of all studies demonstrated a pooled seizure freedom rate of 68.8%. Subject‐level analysis on 18 studies (*n* = 360) demonstrated a significant association between duration of spasms and recurrence of spasms after surgery, with an estimated increased risk of 7% per additional year of spasms prior to operation. Patients who underwent resective surgery that was not a hemispherectomy (i.e., lobectomy, lesionectomy, etc.) had an increased recurrence risk of 57% compared to patients who had undergone hemispherectomy. Resective surgery results in seizure freedom for the majority of pediatric patients with epileptic spasms. Patients who undergo hemispherectomy have lower risk of recurrence than patients who undergo other types of surgical resection. Increased duration of spasms prior to surgery is associated with increased recurrence risk after surgery.

**Plain Language Summary:**

Children with epileptic spasms (ES) that do not respond to medications may benefit from surgical treatment. Our study reviewed existing research to understand how effective surgery is in treating ES in children and what factors predict better outcomes. Researchers followed strict guidelines to search for and analyze studies published since 1985, finding 21 studies with a total of 531 patients. They found that, on average, nearly 70% of children became seizure‐free after surgery. Further individual analysis of 360 patients showed that longer duration of spasms before surgery increased the risk of spasms returning by 7% per year. Additionally, children who had less extensive surgeries, such as removal of only a specific part of the brain, had a 57% higher risk of seizure recurrence compared to those who had a hemispherectomy, which removed or disconnected half of the brain. Overall, the study concludes that surgery can often stop seizures, especially when more extensive surgery is performed and when the surgery is done sooner rather than later.

**Key points**

- The curative utility of surgical resection for refractory epileptic spasms (ES) and prognostic factors related to post‐operative seizure outcome have not been thoroughly studied.
- Our systematic review of 21 studies (*n* = 531) demonstrated a pooled seizure freedom rate of approximately 69% following resective surgery for ES in pediatric patients.
- Subject‐level analysis (*n* = 360) showed significant association between duration of ES and post‐operative recurrence. Patients who underwent hemispherectomy had lower recurrence risk compared to patients who had undergone other types of surgical resection.

## 1 INTRODUCTION

Epileptic spasm (ES) is a distinct form of seizure that requires urgent diagnosis and treatment to optimize patient outcomes. This condition most often presents in early life with an incidence as high as >60 per 100 000 children under the age of 5 years.^1^, ^2^ Additionally, the occurrence of late‐onset spasms has also been observed.^3^ For the purpose of this article, the term “epileptic spasms” will be used to describe spasms occurring both during infancy and beyond, per the International League against Epilepsy (ILAE) recommendations.^4^ Failure to treat ES can lead to profound developmental impairments. Initiation of early treatment and effective seizure reduction can help minimize cognitive and neurological deficits.^5^, ^6^ Therefore, timely evaluation and management are imperative.

Standard first‐line treatments include adrenocorticotropic hormone (ACTH), corticosteroids, and vigabatrin; however, spasms are not always amenable to medical management.^7^ Following the report in 1990 that some cases of ES had a resectable epileptic focus, surgery gradually became an accepted treatment option for intractable spasms.^8^, ^9^ Recent clinical series have reported favorable outcomes following resective surgery for refractory ES, with seizure freedom rates ranging from 42 to 82%, but these studies had small subject populations and varied results.^10^, ^11^ In addition, few studies have examined predictors of favorable long‐term post‐operative results. A comprehensive summary and analysis of post‐operative seizure freedom outcomes are needed to better understand the potentially curative surgical resection for ES on a larger scale.

This systematic review was conducted to (1) evaluate the current literature regarding seizure freedom outcomes after resective surgery for ES in pediatric patients with at least 1 year of post‐operative follow‐up and (2) identify prognostic factors affecting seizure outcomes. The primary outcome measure was complete seizure freedom. To date, this is the first meta‐analysis exploring the post‐operative outcomes of epilepsy surgery in pediatric patients with ES.

## 2 MATERIALS AND METHODS

This study protocol was registered on PROSPERO International Prospective Register of Systematic Reviews (CRD42022322156) prior to commencement. The literature search and study design were conducted according to the Preferred Reporting Items for Systematic Reviews and Meta‐Analysis (PRISMA) guidelines.^12^

### 2.1 Search strategy, study screening, and data extraction

The search strategy was developed by a pediatric neurologist (G.E.) and a medical librarian (AGS.). The search was conducted in March 2022 in PubMed (1809–present), Embase (Embase.com, 1974–present), and Cochrane CENTRAL Register of Controlled Trials (Wiley) databases. The following keywords and database‐specific terminology (e.g., MeSH) were included: ‘epileptic spasm’, ‘infantile spasm’, ‘West syndrome’, ‘drug resistant epilepsy’, ‘epilepsies, partial’, ‘focal epilepsy’, ‘surgical’, ‘surgery’, ‘neurosurgical’, ‘neurosurgery’, ‘resective surgery’, ‘hemispherectomy’, ‘resection’, ‘tuberectomy’, ‘lobectomy’, ‘lesionectomy’, ‘neurosurgical procedures’, ‘anterior temporal lobectomy’, ‘brain surgery’, and ‘temporal lobectomy’. All searches were conducted free of language or date restrictions. Articles without an available English translation were excluded.

Search results from each database were imported into a systematic review software, Covidence (www.covidence.org), and duplicates were removed. The remaining citations were screened by two authors independently (T.K and G.E) through title and abstract review. For the resulting full‐length articles, article relevance, suitability, and quality were evaluated by T.K and G.E. using predetermined eligibility criteria. Disagreements were resolved by consensus between two authors.

We included full‐length peer‐reviewed original research articles that described seizure outcomes measured using Engel's or ILAE classification for at least five pediatric patients (<18 years of age) who underwent resective surgery for ES. Exclusion criteria included: (1) conference abstracts, case reports, case series with fewer than five subjects, literature reviews, systematic reviews, editorials, letters to the editor; (2) articles without an English translation; (3) lack of pediatric ES patients; (4) inability to isolate outcomes of ES patients; (5) lack of information on seizure frequency reduction after intervention; (6) patients who had undergone palliative surgical procedures (e.g., VNS, corpus callosotomy); and (7) <1 year of follow‐up. For studies with potentially overlapping patient populations, only the most recent article was included.

Data extraction was completed using a standardized data collection form. The following data were extracted from each study: study details (title, author, year of publication, location, study design, sample size, date range), total number of participants, subject demographics (age, age at ES onset, age at surgery, sex), spasms etiology and indications for surgery, MRI findings, types of resective surgery, follow‐up duration, and outcomes. The main outcome was seizure freedom following resective surgery. Complete seizure freedom is defined as the absence of disabling seizures, commonly reported as Engel Class I or ILAE Class I.^13^, ^14^

For studies that included subject‐level data, the following data were extracted as available: age at ES onset, seizure type(s), MRI findings, etiology, age at surgery, surgery type, follow‐up time, and seizure reduction outcome. The primary outcome of interest was seizure freedom rate at the last follow‐up to best evaluate long‐term seizure reduction. For studies that did not include subject‐level data, we reached out to the authors to request it. Barba et al.^15^ and Erdemir et al.^16^ kindly provided the missing information.

### 2.2 Statistical analysis

For study‐level analysis, the rates of seizure freedom were pooled from all papers included in the study. The heterogeneity among studies was assessed using Q statistics and I^2^ was used to determine the magnitude of heterogeneity. Statistically significant heterogeneity was considered Q statistic *p* < 0.05 and I^2^ ≥ 50%. Fixed or random effects models were used to calculate the pooled estimate of favorable seizure freedom outcomes and 95% confidence intervals (CIs), depending on the degree of heterogeneity. Studies were separated into subgroups according to age at ES onset (<1 year old and >1 year old), duration of spasms (<2 years, >2 years), and age at surgery (<2 years old, 2–5 years old, >5 years old), and pooled seizure freedom was compared across subgroups using the same method. Publication bias was assessed graphically using a funnel plot and statistically using Begg^17^ and Egger^18^ tests. R Studio Version 2023.06.0+421 was used for study‐level analyses.

For subject‐level analysis, descriptive analyses of characteristics of subjects were performed. Continuous variables were reported as means (standard deviations), and categorical variables were reported as frequencies (percentages). Cox proportional hazard models were used to examine the relationship between each of the risk factors and the seizure recurrence as the endpoint variable after the surgery. Multivariable Cox proportional hazards model was conducted using the forward selection method with an entry level of 0.1. Subject‐level analyses were performed using SAS (v. 9.4, SAS Institute, Inc., Cary, NC) with a type I error of 0.05.

## 3 RESULTS

**FIGURE 1:**
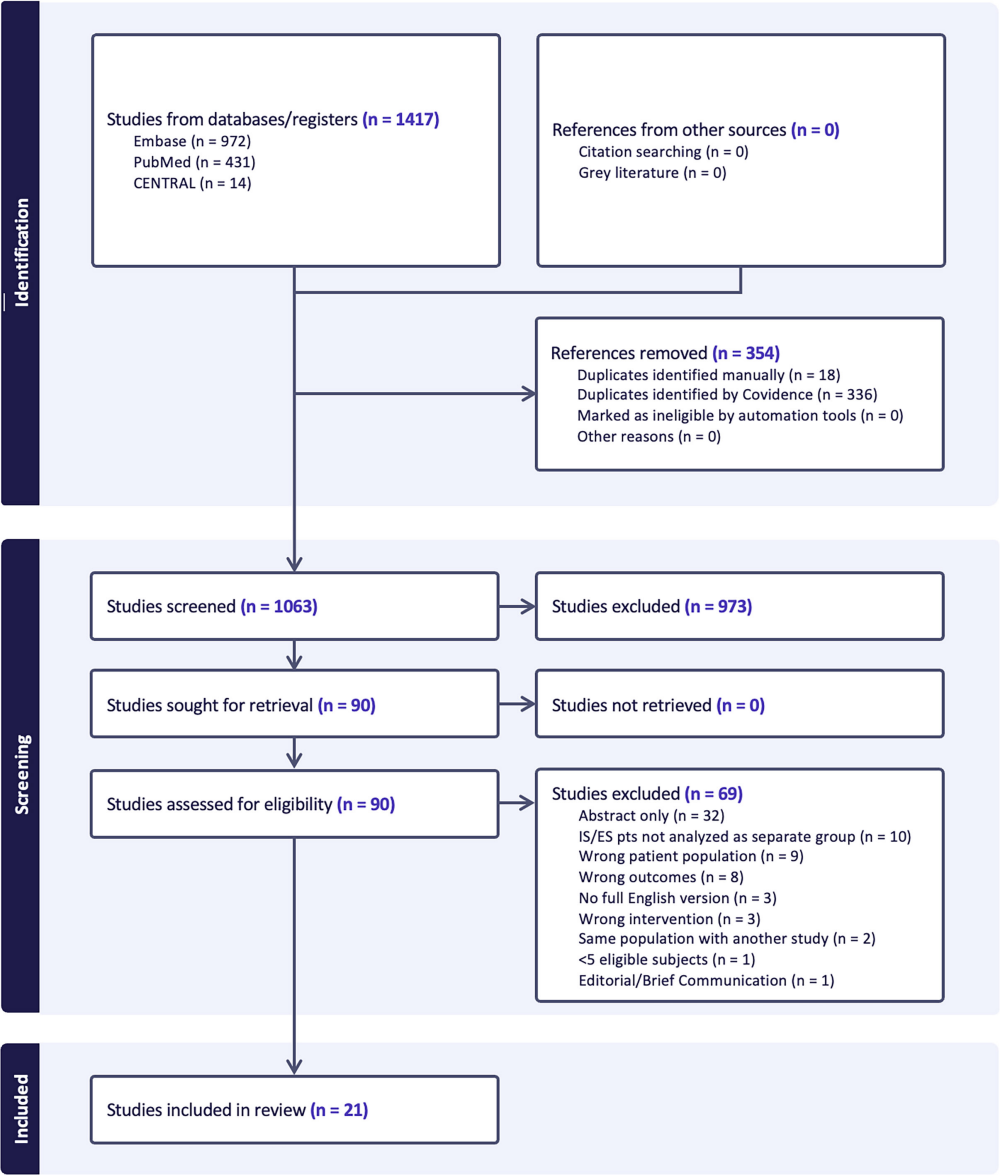
PRISMA flow diagram depicting search and review strategy.

Figure 1 displays the results of the search and review process using the PRISMA flow diagram.^19^ A total of 531 patients from 21 studies met the eligibility criteria.

Table 1 provides a summary of the 21 studies included in the systematic review, with pooled seizure freedom rates and average values for patient age at surgery, MRI findings, use of intraoperative EEG, duration of ES, and follow‐up time. Publication dates ranged from 1993 to 2021 and all articles described retrospective clinical studies using institutional medical records. Of the 21 publications, nine were conducted in Asia, six were conducted in North America, four were conducted in Europe, one was conducted in South America, and one was conducted in Australia. All 21 articles were available in English.

**TABLE 1:** Summary of included studies.

Study No.	Authors & Year	Journal	Study origin	Patient age at surgery (years)^a^	Duration of spasms prior to surgery (years)^a^	Follow‐up time (years)^a^	Total number of patients	Use of intraoperative EEG (*n*, %)	Negative MRI findings (*n*, %)	Seizure‐free patients
1	Barba et al., 2017^15^	Ann. Clin. Transl. Neurol.	Italy	5.8	4.5	5.7	80	—	3 (3.75)	49 (61.3%)
2	Caraballo et al., 2016^41^	Seizure	Argentina	1.8	1.5	5.5	6	—	—	6 (100%)
3	Chipaux et al., 2017^23^	Seizure	France	4.6	3.4	7.2	60	—	—	45 (75%)
4	Chugani et al., 2015^7^	Epilepsia	United States	5.1	4.0	3.8	65	65 (100)	18 (27.7)	46 (70.8%)
5	Chugani et al., 1993^42^	Epilepsy Res.	United States	1.6	1.6	2.2	23	23 (100)	13 (56.5)	14 (60.9%)
6	Erdemir et al., 2021^16^	Epilepsy Res.	United States	1.9	1.3	4.7	70	—	—	42 (60%)
7	Hur et al., 2010^43^	Brain Dev.	Switzerland	4.6	4.0	2.7	9	—	8 (88.9)	7 (77.8%)
8	Inage et al., 2012^44^	Brain Dev.	Netherlands	NA	NA	2.9	11	11 (100)	3 (27.3)	7 (63.6%)
9	Iwatani et al., 2012^45^	Pediatr. Neurosurg.	Netherlands	1.4	1.0	4.9	6	—	—	4 (66.7%)
10	Kang et al., 2005^46^	Pediatr. Neurosurg.	Korea	2.8	2.5	2.7	11	—	2 (18.2)	8 (72.7%)
11	Lee et al., 2014^47^	Epileptic Disord.	Korea	1.9	1.4	2.7	15	—	—	9 (60%)
12	Liu et al., 2021^11^	Epilepsy Res.	France	3.3	2.4	3.1	64	61 (95.3)	11 (17.2)	53 (82.8%)
13	Liu et al., 2012^20^	Epilepsia	Netherlands	4.2	3.3	3.0	17	17 (100)	—	11 (64.7%)
14	Maton et al., 2008^48^	Neuropediatrics	United States	1.4	1.1	7.3	6	—	—	4 (66.7%)
15	Metsahonkala et al., 2015^49^	J. Neurosurg. Pediatr.	Germany	9.8	8.1	2.7	6	5 (83.3)	1 (16.7)	4 (66.7%)
16	Mohamed et al., 2011^50^	J. Child Neurol.	United States	5.5	5.1	4.3	10	—	—	6 (60%)
17	Moseley et al., 2012^51^	Seizure	United States	3.3	2.2	3.6	11	—	1 (9)	6 (54.5%)
18	Taussig et al., 2012^21^	J. Neurosurg. Pediatr.	France	2.3	1.9	3.2	11	11 (100)	—	8 (72.7%)
19	Xu et al., 2019^10^	Epilepsy Res.	United States	8.5	4.3	3.1	26	26 (100)	5 (19.2)	11 (42.3%)
20	Yu et al., 2015^52^	Epilepsy Res.	Netherlands	10.4	6.9	1.6	19	19 (100)	—	12 (63.2%)
21	Yum et al., 2011^22^	Clin. Neurol. Neurosurg.	Netherlands	1.6	1.1	6.0	5	—	—	4 (80%)

Abbreviations: —, information not reported in article; NA, not available.

^a^ Mean or median, depending on which measure was provided by the study.

Across all 21 included studies, median ages at ES onset and surgery were 0.79 years (mode = 0.45 years) and 3.32 years (mode = 2 years), respectively. Median follow‐up duration was 3.22 years (mode = 3 years).

Presurgical evaluations exhibited variability across studies. Typically, patients underwent scalp video EEG, MRI, and PET. In 10 studies, invasive EEG (stereo EEG or subdural grids) was employed. Ictal SPECT was part of the presurgical evaluation in eight studies, while MEG was used in three studies. Additionally, one study integrated DTI with other presurgical tests.

All 21 studies provided detailed information about the presence of other seizure types, etiology of epileptic spasms, MRI findings, and the surgery type. The most commonly reported etiologies were focal cortical dysplasia (FCD), tumor‐related, and tuberous sclerosis (TSC). Articles described various surgical approaches, including lesionectomy, multilobar resections, lobectomy, hemispherectomy, and hemispherectomy.

Out of the reviewed literature, only five studies reported post‐surgical complications.^7^, ^15^, ^20^, ^21^, ^22^ The largest of these studies, Barba et al.,^15^ reported that 15 out of 80 patients (18%) experienced post‐operative complications, with subgaleal fluid collection and hydrocephalus being the most common. Chugani et al.^7^ reported that 15 out of 65 patients (23%) experienced post‐operative complications, with hydrocephalus being the most common (73% of all complications). Hydrocephalus was seen more commonly in patients who had undergone hemispherectomy. The other three studies reported post‐operative complications at varying rates: 41% (7/17 patients), 50% (5/10 patients), and 60% (3/5 patients).^20^, ^21^, ^22^ Unlike the studies by Barba et al.^15^ and Chugani et al.,^7^ which focused solely on surgery‐related complications, these studies also included both permanent and temporary neurological deficits, possibly accounting for the higher complication rates.

### 3.1 Pooled seizure freedom rate – study‐level analysis

**FIGURE 2:**
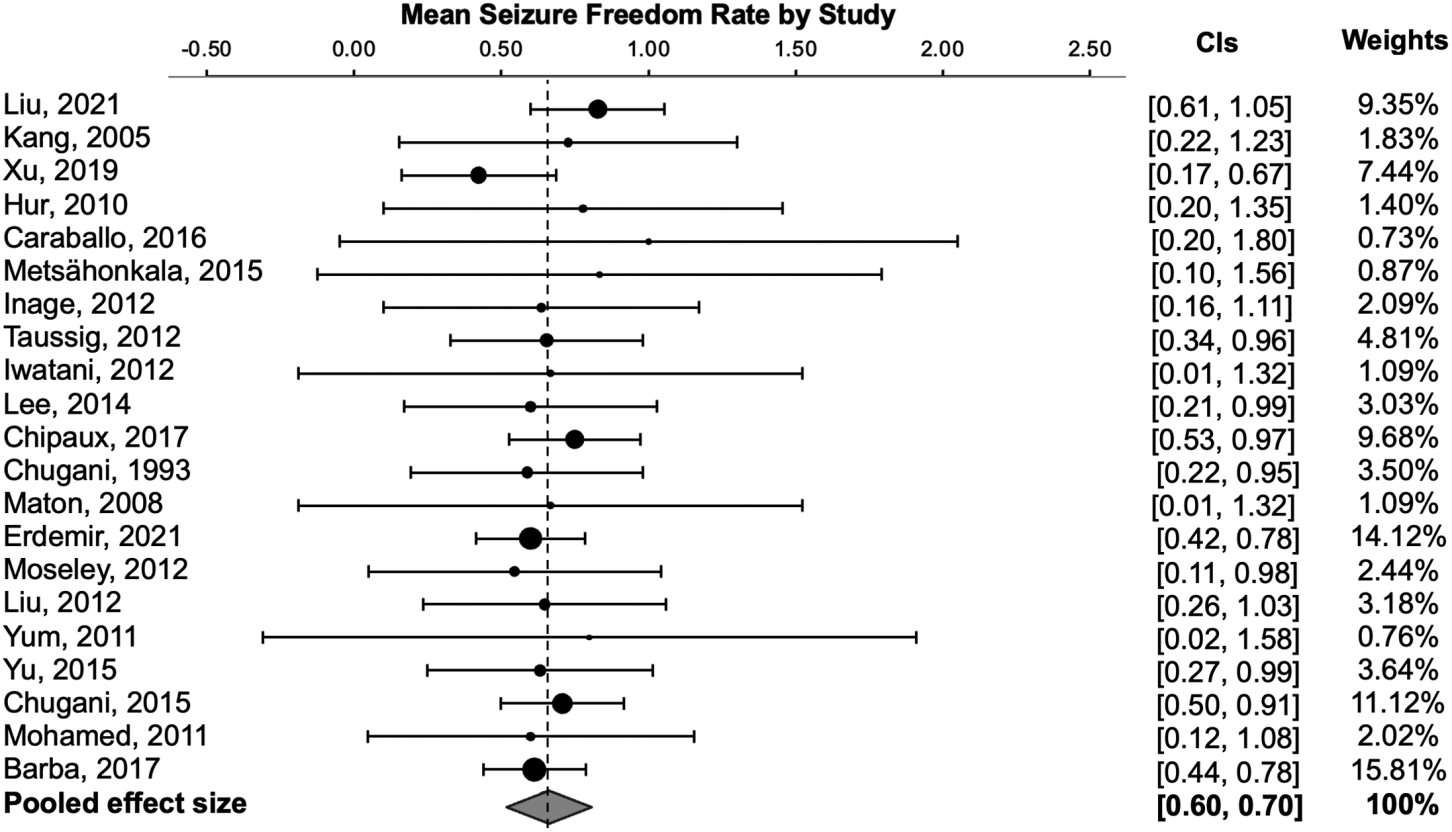
Seizure freedom rates across all included studies.

**FIGURE 3:**
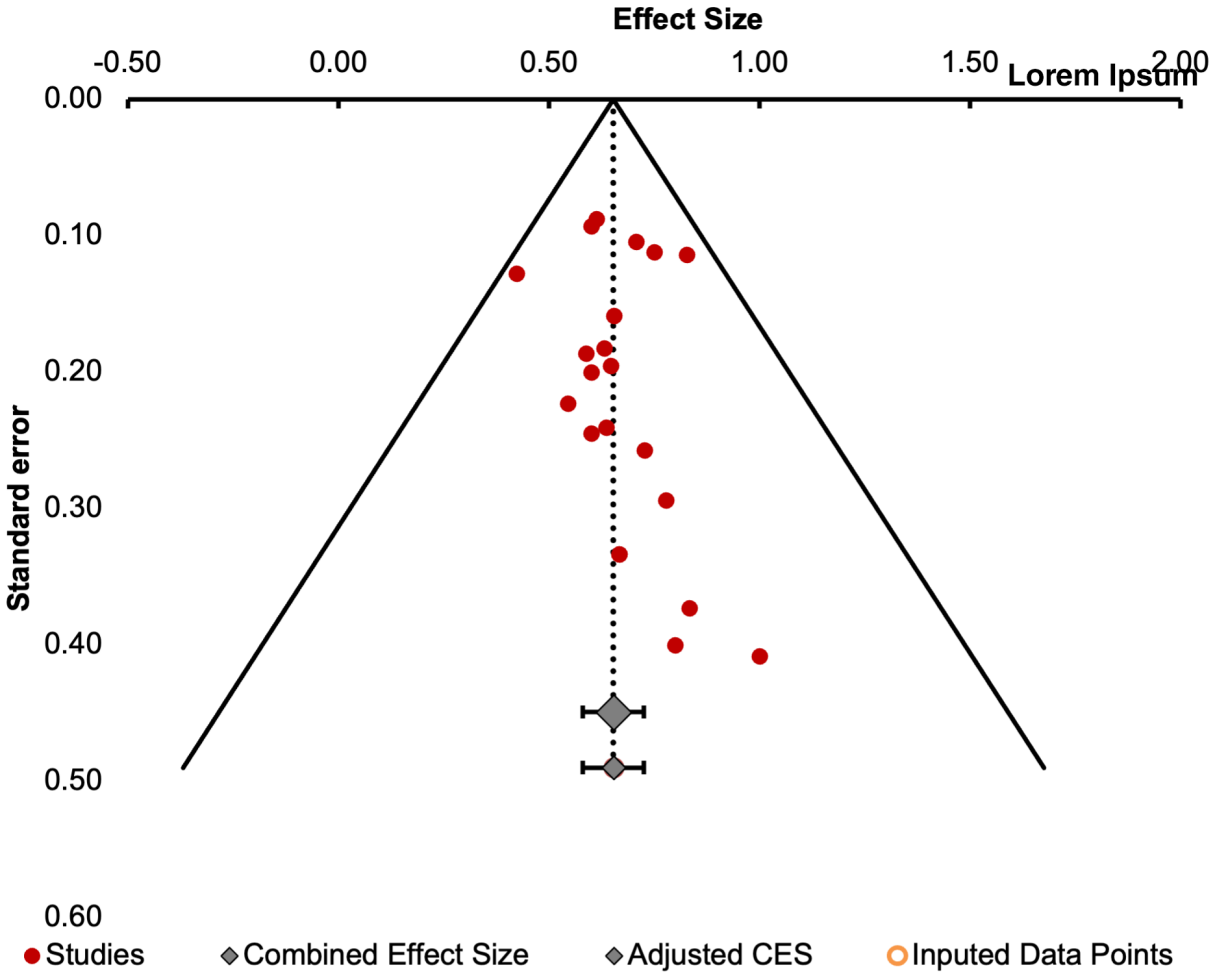
Funnel plot depicting publication bias of the 21 included studies.

The pooled rate of post‐operative seizure freedom (effect size) from the 21 studies was 68.79% (SE = 2.38, 95% CI [64.11–73.46]) (Figure 2). Using a random effect model, we found low heterogeneity across studies (Q = 30.39, I^2^ = 17.73%). There was no evidence of publication bias for the estimate of seizure freedom rate using visual inspection of the funnel plot (Figure 3) or the Egger regression test (*p* > 0.05). However, the Begg rank test did demonstrate significant correlation between the rank of effect sizes and corresponding sampling variances, indicating that our results could be affected by publication bias (*p* = 0.03).

**FIGURE 4:**
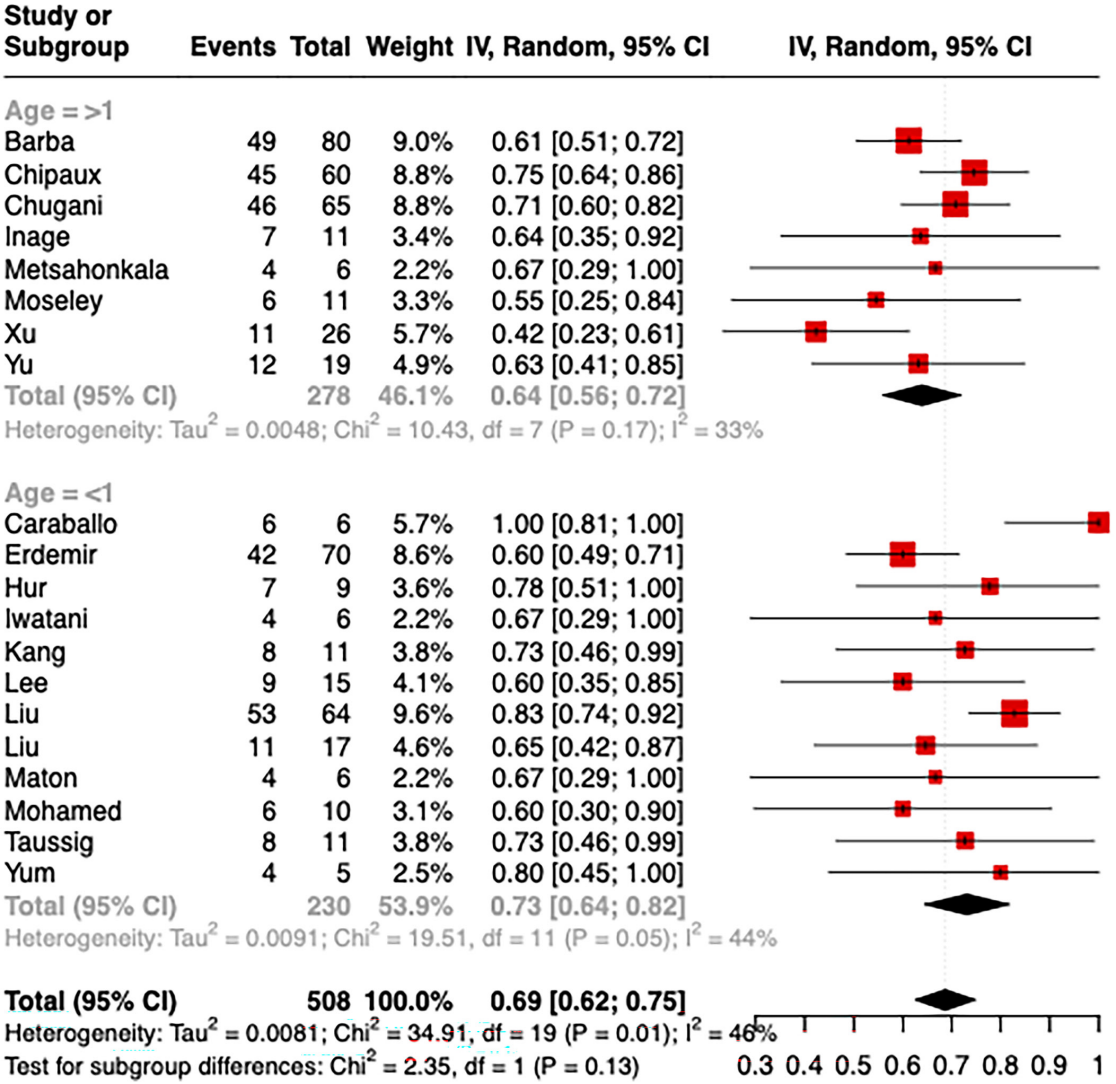
Seizure freedom rates separated by studies reporting median age at seizure onset below and over 1 year old.

**FIGURE 5:**
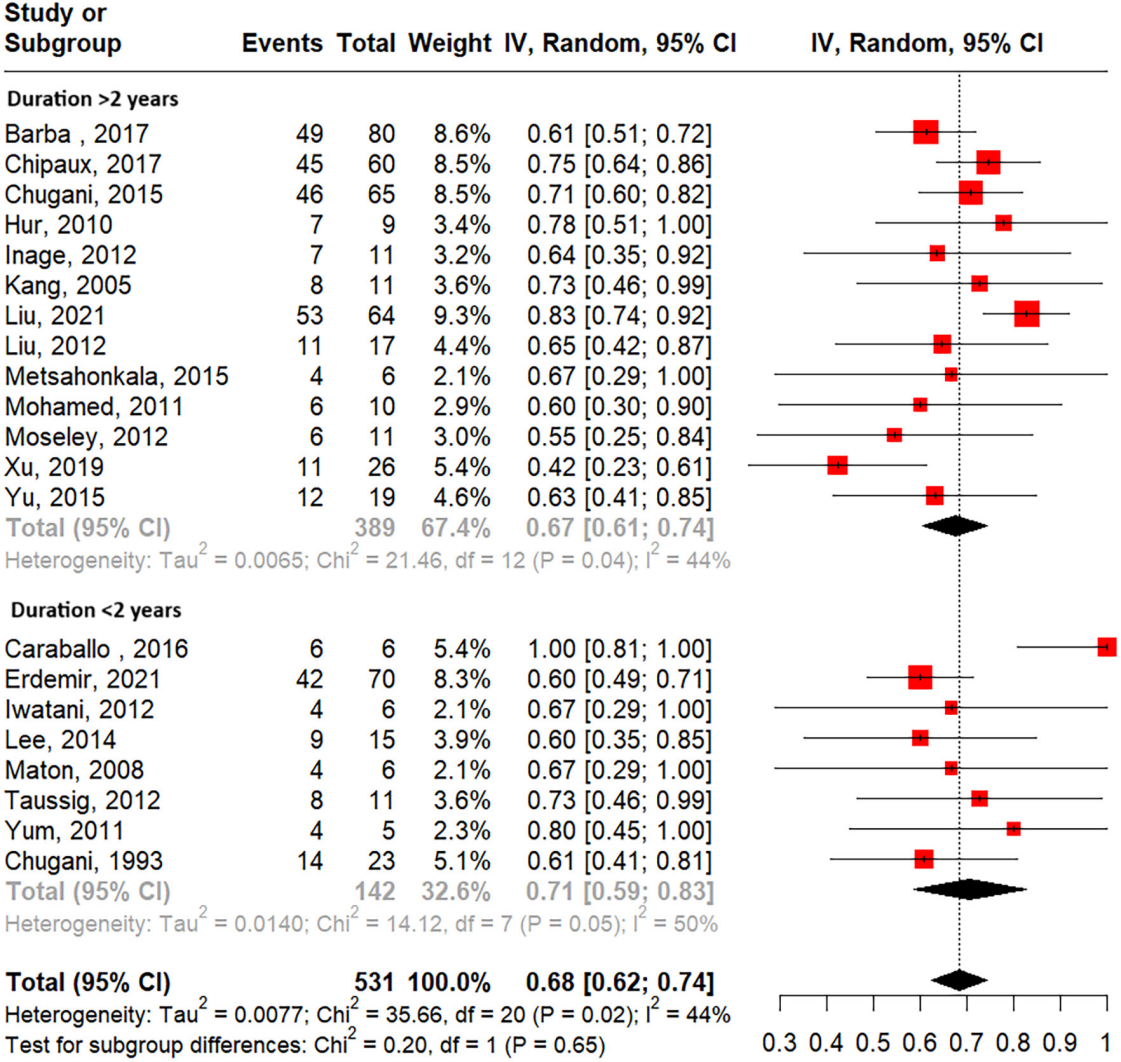
Seizure freedom rates separated by studies reporting median spasm duration of less than or greater than 2 years old.

**FIGURE 6:**
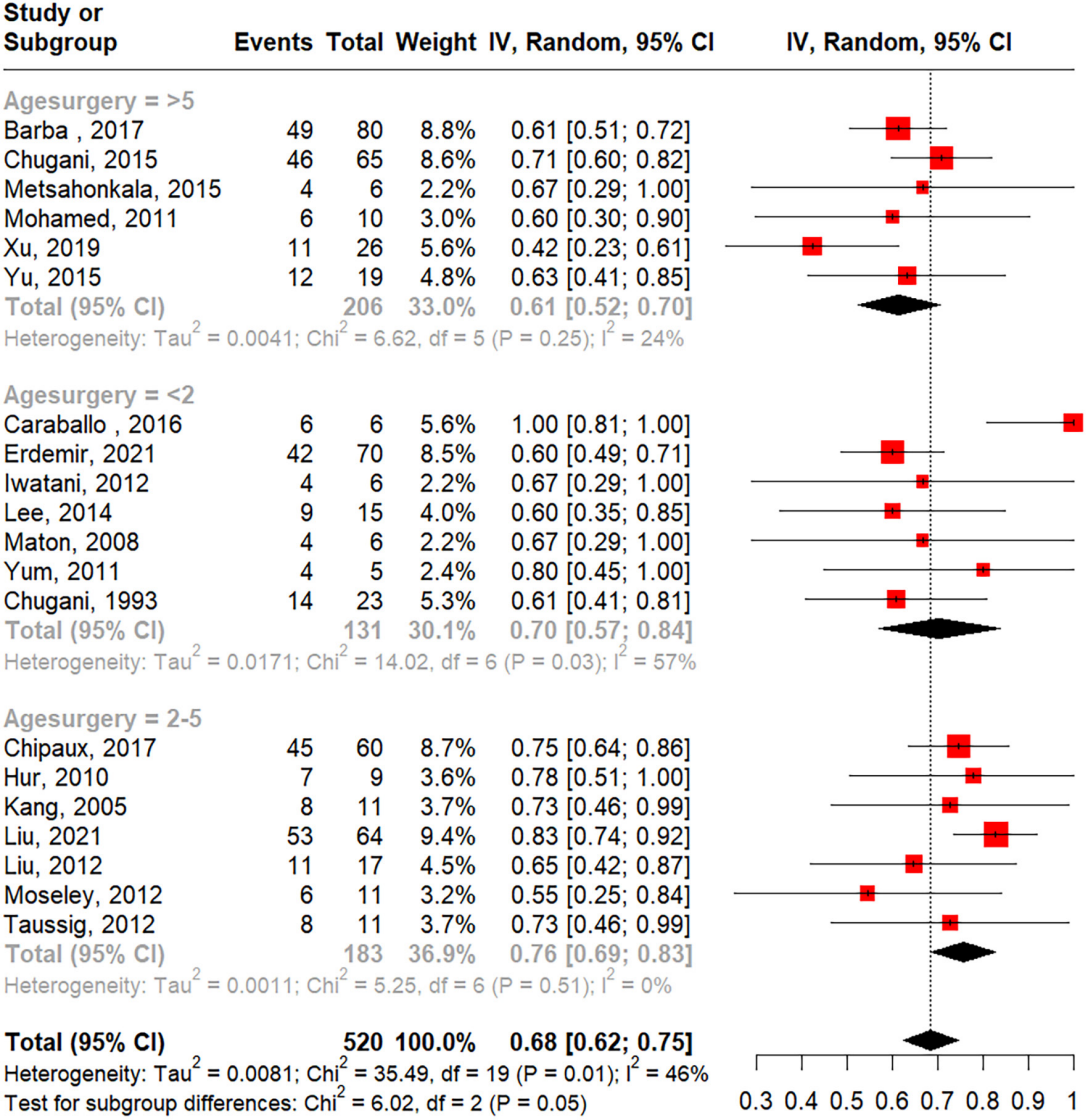
Seizure freedom rates separated by studies reporting median age at surgery <2 years, between 2 and 5 years, and >5 years old.

Subgroup analysis showed a higher pooled seizure freedom rate in studies with an average epilepsy onset before 1 year old (73%) compared to those with an average onset age older than 1 year (64%) (Figure 4), though this difference was not statistically significant. The pooled seizure freedom rate showed no significant difference when studies were categorized by epileptic spasm duration of <2 years versus at least 2 years (Figure 5) or by age at the time of surgery younger than 5 years versus older than 5 years (Figure 6).

### 3.2 Factors predicting seizure freedom – subject‐level analysis

**FIGURE 7:**
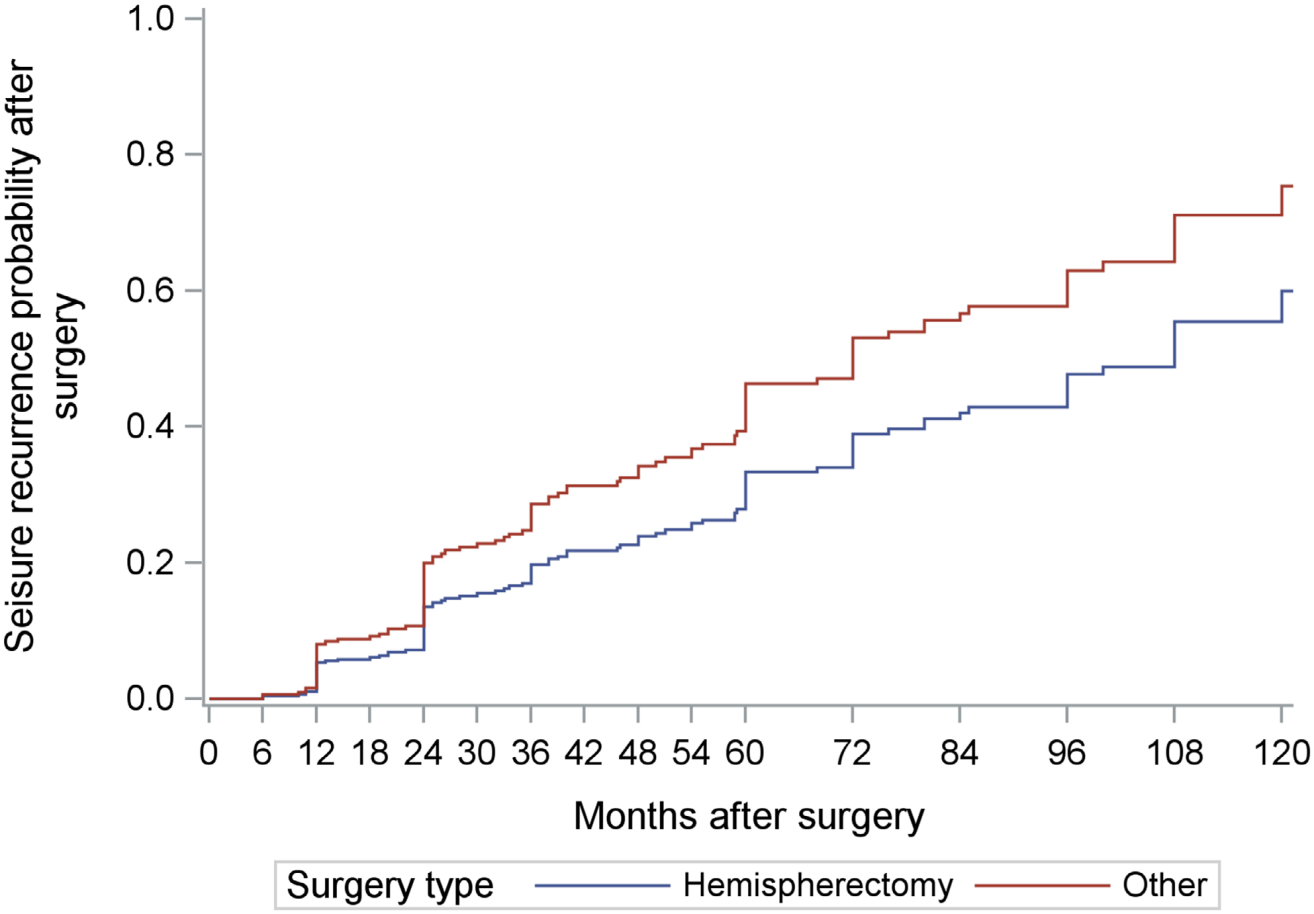
Seizure risk recurrence over time (months after surgery) separated by patients who underwent hemispherectomy versus other types of resective surgery.

Of the 21 included studies, 18 studies provided subject‐level data, with a total of 360 patients (Table 2). Subject‐level information on age at onset, duration of ES, age at surgery, MRI findings, etiology, surgery type, follow‐up time, and outcome can be found in Table S1. Duration of ES was found to have a significant association with recurrence of spasms after surgery, with an estimated increased risk of 7% per additional year of ES (HR 1.07, 95% CI 1.03–1.12, *p* = 0.003). Surgery type was also significantly associated with recurrence of spasms. Patients who had undergone resective surgery that was not a hemispherectomy (i.e., lobectomy, lesionectomy, etc.) had an estimated increased recurrence risk of 57% compared to patients who had undergone hemispherectomy (HR 1.57, 95% CI 1.03–2.31, *p* = 0.04). The difference in recurrence risk between hemispherectomy and other types of resective surgeries increased with time after surgery (Figure 7).

**TABLE 2:** Descriptive summary from subject‐level analysis.

Variable	Subjects (*N*, %)
	Total: *N* = 360
Age at onset (SD), months	16.5 (24.09)
Duration of spasms (SD), years	3.5 (3.72)
Months after surgery (SD)	46.3 (38.87)
Seizure type	
Spasms only	218 (60.56%)
Spasms and Focal Seizures	130 (36.11%)
Spasms and Generalized Seizures	12 (3.33%)
Surgery type	
Hemispherectomy	95 (26.39%)
Other (non‐hemispherectomy)	265 (73.61%)
MRI Findings	
Positive	308 (85.56%)
Negative	52 (14.44%)

Abbreviations: *N*, number of subjects; SD, standard deviation.

Out of 340 patients, 50 had a known genetic etiology. Among these 50 patients, 29 achieved seizure freedom (58%). Of the 50 patients with a genetic etiology, 42 had tuberous sclerosis complex, and 24 of these patients achieved seizure freedom (57%).

Younger age at onset, positive MRI findings, and presence of accompanying seizures (e.g. focal seizures, generalized seizures) were not significantly associated with increased or decreased risk of recurrence. Complete results of univariate and multivariate Cox proportional hazards analysis of other factors including age at onset, MRI findings, and seizure types are provided in Table 3.

**TABLE 3:** Univariate and multivariate cox proportional hazards analysis with seizure freedom as outcome.

Analysis	Variable	*p*‐Value	Hazard ratio	95% CI
Univariate	Age at onset (months)	0.0664	1.006	1, 1.013
	Duration of epilepsy (years)	0.0015	1.070	1.026, 1.116
	Positive MRI	0.1417	0.709	0.449, 1.122
	Spasms + Focal Seizures	0.1286	1.303	0.926, 1.832
	Spasms + Generalized Seizures	0.1811	1.646	0.793, 3.415
	Hemispherectomy no vs. yes	0.0426	1.538	1.025, 2.307
Multivariate	Duration of epilepsy (years)	0.0029	1.072	
	Hemispherectomy no vs. yes	0.0391	1.569	

## 4 DISCUSSION

In the present study, we performed a systematic review and meta‐analysis to identify the rate and predictors of seizure freedom in pediatric patients with epileptic spasms who undergo resective surgery. Our meta‐analysis examining 21 studies and total of 531 patients indicates that 68.8% of these patients achieved excellent outcomes, defined as being seizure‐free for at least 12 months following surgery. These results are consistent with the findings from five previous largest series on epilepsy surgery in children with epileptic spasms, where post‐operative seizure freedom rates ranged from 60% to 83%.^7^, ^11^, ^15^, ^16^, ^23^ These findings demonstrate that epilepsy surgery has a favorable outcome in carefully selected pediatric patients with epileptic spasms. Traditionally, such patients were not considered for surgery due to non‐localizing EEG changes and symmetric epileptic spasms. However, the paradigm has gradually shifted over the last three decades, and epilepsy surgery is now widely accepted as a viable treatment option for cases with resectable brain lesions.^24^

Our subject‐level analysis of 360 patients from 18 studies revealed that the likelihood of achieving a favorable outcome is even higher for patients with shorter duration of spasms. Each additional year spent living with epileptic spasms, the risk of recurrence increased by approximately 7% in our sample. Previous studies consistently showed the benefits of early surgery, yielding improved outcomes across both adult and pediatric populations with various seizure types.^15^, ^25^, ^26^, ^27^, ^28^ Pelliccia et al. showed that even non‐drug‐resistant patients who underwent epilepsy surgery are more likely to have favorable outcomes.^29^ We know that some patients with epileptic spasms, especially those with lesional etiology, have higher risk of having drug‐resistant spasms.^30^ Therefore, it is important to think about surgery earlier in the course of the disease, as this can lead to better seizure and developmental outcomes for these patients. Delay in the treatment of spasms has been shown to be detrimental to children's development.^31^, ^32^

Unfortunately, despite national and international guidelines advocating for prompt referral, many children with drug‐resistant epilepsy still face delayed surgical evaluation.^33^ Recent research by Buttle et al. examined the attitudes of child neurologists practicing in North America, reporting that 80% of surveyed pediatric neurologists stated that they would be reluctant to refer patients with generalized EEG findings or generalized seizures for surgical evaluation.^34^ Although spasm semiology and EEG findings are not always helpful to localize the lesion for patients with epileptic spasms, this should not deter patients from being considered for epilepsy surgery. A concerted effort to raise awareness among healthcare professionals about the potential benefits of early surgery for epileptic spasms is needed. Encouraging open discussions between healthcare providers and parents/caregivers about the advantages and risks of surgical intervention can empower families to make informed decisions for their child's well‐being. Our results provide much needed data for routine presurgical patient counseling.

Our study also showed that the patients who underwent hemispherectomy experienced a lower risk of seizure recurrence compared to those who underwent smaller resections. This result aligns with findings from previous meta‐analyses investigating predictors of surgical outcomes in pediatric epilepsy surgery patients.^35^, ^36^ Emphasizing the importance of complete resections in epilepsy surgery, historical practices often favored incomplete resections in an attempt to minimize neurological deficits. However, prior retrospective studies consistently indicate that incomplete resection of the epileptogenic zone is linked to higher rates of seizure recurrence, often necessitating a second surgery.^37^, ^38^ For some families, the failure of the initial incomplete surgery may act as a deterrent to pursuing a second surgery. The inclination toward larger resections diminishes the likelihood of incomplete resections, explaining the favorable outcomes associated with hemispherectomy. It is also noteworthy that, although only a limited number of studies reported post‐surgical complications, hydrocephalus was identified as the most common complication and was more frequently observed following hemispherectomy procedures.

While the presence of a lesion on MRI has been independently associated with a positive outcome,^7^ our study did not find a correlation between the presence of a lesion and the overall outcome. This discrepancy may be attributed to the relatively small size of our normal MRI group or the utilization of pre‐surgical tools such as PET, SPECT, and intracranial EEG.

Additionally, genetic factors may play a crucial role in predicting surgical success. Recent literature recommends evaluating children with drug‐resistant epilepsy due to genetic etiologies for epilepsy surgery, although seizure freedom rates are lower than in patients with non‐genetic etiologies.^39^ Our study also found that seizure freedom rates were lower in patients with genetic etiologies compared to non‐genetic patients. However, the number of patients with genetic epilepsies in our study was small, and the population was not heterogeneous, as it was predominantly composed of children with tuberous sclerosis. For patients with tuberous sclerosis and drug‐resistant epilepsy, epilepsy surgery can be beneficial in certain settings. Detecting epileptic tubers during pre‐surgical evaluation is crucial for these patients.^40^ Although tuberous sclerosis is strongly associated with infantile spasms, there is growing evidence that other genetic mutations associated with epileptic spasms can influence treatment outcomes. Future research should consider the genetic profiles of patients undergoing epilepsy surgery to better understand how specific genetic mutations may impact the likelihood of achieving seizure freedom.

Our study has certain limitations. The limited data and retrospective nature of the included studies should be heavily considered when interpreting the results of this review. Retrospective studies are inherently limited by their design and prospective research would be ideal. Not all studies included patient‐level data, so some could not be used in the more granular analysis, and not all studies that included patient‐level data provided the same parameters. In addition, populations varied across studies with regard to age group, etiology, and disease severity, so the findings should be evaluated with some caution.

In conclusion, resective surgery for epileptic spasms in children results in favorable seizure outcomes, with a seizure‐freedom rate of approximately 69%. Longer duration of spasms and lobectomy or lesionectomy are relative risk factors for post‐operative recurrence of spasms; hemispherectomy and earlier surgical intervention may lead to favorable outcomes in children with epileptic spasms. However, given the limitations inherent to the present study, further research should be conducted in a prospective, multi‐institutional manner to explore seizure freedom rates and prognostic indicators in a larger population. In addition to the risk factors studied in our systematic review, other factors, including genetic etiologies, developmental outcomes, educational attainment, and quality of life, should be examined.

## AUTHOR CONTRIBUTIONS

Study conception: Gozde Erdemir and Taylor Kolosky. Data collection: Taylor Kolosky and Andrea Goldstein Shipper. Data analysis and interpretation: Soren Bentzen, Gozde Erdemir, Taylor Kolosky, Busra Tozduman, and Kai Sun. Manuscript composition: Taylor Kolosky, Gozde Erdemir, Kai Sun, Ahsan N. Moosa. Critical review of manuscript: all authors.

## CONFLICT OF INTEREST STATEMENT

No authors have any conflicts of interest to disclose. We confirm that we have read the Journal's position on issues involved in ethical publication and affirm that this report is consistent with those guidelines.

## Supporting information


Table S1.

## Data Availability

Individual study data can be made available on request to tkolosky@som.umaryland.edu.
