# Supplementary material for: Epilepsy surgery for children with epileptic spasms: A systematic review and meta‐analysis with focus on predictors and outcomes
Source: Epilepsia Open. 2024 Jul 2;9(4):1136–47. doi: 10.1002/epi4.13007 (PMC11296110; doi:10.1002/epi4.13007)
Supplement: Supplementary file 1 — Table S1. [file EPI4-9-1136-s001.docx]

Supplemental Table 1 - Additional data from subject-level analysis

| **Study No.** | **Subject No.** | **MRI +/-** | **Etiology** | **Age at onset (mo.)** | **Duration of epilepsy (mo.)** | **Age at surgery (mo.)** | **Surgery type** | **Follow-up time (mo. after surgery)** | **Seizure free?** |
| --- | --- | --- | --- | --- | --- | --- | --- | --- | --- |
| 1 | 1.01 | + | structural | 4.8 | 139.2 | 144 | lesionectomy | 96 | Y |
| 1 | 1.02 | + | structural | 60 | 30 | 90 | multilobar | 55.2 | N |
| 1 | 1.03 | + | structural | 1.8 | 107.4 | 109.2 | other resective surgery | 14.4 | N |
| 1 | 1.04 | + | structural | 36 | 6 | 42 | lesionectomy | 72 | Y |
| 1 | 1.05 | + | structural | 1.44 | 63.36 | 64.8 | multilobar | 45.6 | N |
| 1 | 1.06 | + | structural | 2.4 | 10.8 | 13.2 | lesionectomy | 69.6 | Y |
| 1 | 1.07 | + | structural | 3.6 | 138 | 141.6 | multilobar | 118.8 | Y |
| 1 | 1.08 | + | structural | 24 | 45.6 | 69.6 | lesionectomy | 171.6 | Y |
| 1 | 1.09 | + | structural | 0.48 | 6.72 | 7.2 | lesionectomy | 127.2 | N |
| 1 | 1.10 | + | structural | 6 | 42 | 48 | other resective surgery | 12 | N |
| 1 | 1.11 | + | structural | 3 | 21 | 24 | other resective surgery | 24 | N |
| 1 | 1.12 | + | structural | 3.6 | 8.4 | 12 | multilobar | 12 | N |
| 1 | 1.13 | + | structural | 2.4 | 165.6 | 168 | other resective surgery | 24 | Y |
| 1 | 1.14 | + | structural | 4.8 | 87.6 | 92.4 | lesionectomy | 13.2 | Y |
| 1 | 1.15 | + | structural | 6 | 18 | 24 | other resective surgery | 10.8 | N |
| 1 | 1.16 | + | structural | 18 | 36 | 54 | lesionectomy | 68.4 | Y |
| 1 | 1.17 | + | structural | 6 | 6 | 12 | multilobar | 6 | N |
| 1 | 1.18 | + | structural | 0.96 | 73.44 | 74.4 | other resective surgery | 25.2 | Y |
| 1 | 1.19 | + | structural | 0.96 | 7.44 | 8.4 | multilobar | 15.6 | Y |
| 1 | 1.20 | + | structural | 9.6 | 14.4 | 24 | lesionectomy | 144 | Y |
| 1 | 1.21 | + | structural | 48 | 60 | 108 | multilobar | 60 | N |
| 1 | 1.22 | + | structural | 6 | 150 | 156 | lesionectomy | 48 | N |
| 1 | 1.23 | + | structural | 8.4 | 115.2 | 123.6 | lesionectomy | 58.8 | N |
| 1 | 1.24 | + | structural | 6 | 6 | 12 | other resective surgery | 96 | N |
| 1 | 1.25 | + | structural | 2.4 | 79.2 | 81.6 | multilobar | 9.6 | Y |
| 1 | 1.26 | + | inflammatory | 60 | 58.8 | 118.8 | multilobar | 49.2 | Y |
| 1 | 1.27 | + | structural | 32.4 | 75.6 | 108 | lesionectomy | 60 | N |
| 1 | 1.28 | + | structural | 25.2 | 10.8 | 36 | other resective surgery | 36 | N |
| 1 | 1.29 | + | structural | 6 | 12 | 18 | lesionectomy | 40.8 | Y |
| 1 | 1.30 | + | structural | 36 | 58.8 | 94.8 | multilobar | 58.8 | N |
| 1 | 1.31 | + | structural | 19.2 | 88.8 | 108 | lesionectomy | 120 | Y |
| 1 | 1.32 | + | structural | 48 | 75.6 | 123.6 | hemispherectomy | 12 | N |
| 1 | 1.33 | + | structural | 9 | 102.6 | 111.6 | multilobar | 12 | N |
| 1 | 1.34 | + | structural | 3.6 | 80.4 | 84 | lesionectomy | 60 | N |
| 1 | 1.35 | + | structural | 4.8 | 79.2 | 84 | multilobar | 180 | N |
| 1 | 1.36 | + | structural | 1.2 | 142.8 | 144 | multilobar | 12 | Y |
| 1 | 1.37 | + | structural | 6 | 42 | 48 | other resective surgery | 12 | N |
| 1 | 1.38 | + | structural | 8.4 | 111.6 | 120 | lesionectomy | 84 | Y |
| 1 | 1.39 | + | structural | 16.8 | 31.2 | 48 | multilobar | 60 | N |
| 1 | 1.40 | + | structural | 1.2 | 118.8 | 120 | hemispherectomy | 12 | Y |
| 1 | 1.41 | + | structural | 3.6 | 32.4 | 36 | lesionectomy | 60 | Y |
| 1 | 1.42 | + | structural | 6 | 6 | 12 | other resective surgery | 24 | N |
| 1 | 1.43 | + | structural | 4.8 | 67.2 | 72 | lesionectomy | 24 | N |
| 1 | 1.44 | + | structural | 3.6 | 8.4 | 12 | other resective surgery | 60 | N |
| 1 | 1.45 | + | structural | 8.4 | 39.6 | 48 | other resective surgery | 24 | N |
| 1 | 1.46 | + | structural | 3.6 | 20.4 | 24 | other resective surgery | 10.8 | N |
| 1 | 1.47 | - | structural | 4.8 | 19.2 | 24 | lesionectomy | 72 | Y |
| 1 | 1.48 | + | structural | 1.2 | 22.8 | 24 | other resective surgery | 24 | Y |
| 1 | 1.49 | + | structural | 6 | 30 | 36 | other resective surgery | 24 | N |
| 1 | 1.50 | + | structural | 12 | 48 | 60 | hemispherectomy | 48 | N |
| 1 | 1.51 | + | structural | 6 | 54 | 60 | other resective surgery | 12 | N |
| 1 | 1.52 | + | structural | 12 | 84 | 96 | lesionectomy | 12 | N |
| 1 | 1.53 | + | structural | 10.8 | 25.2 | 36 | multilobar | 60 | N |
| 1 | 1.54 | + | structural | 6 | 18 | 24 | lesionectomy | 156 | N |
| 1 | 1.55 | + | structural | 3.6 | 140.4 | 144 | lesionectomy | 24 | Y |
| 1 | 1.56 | + | structural | 84 | 36 | 120 | lesionectomy | 96 | N |
| 1 | 1.57 | + | structural | 3.6 | 32.4 | 36 | lesionectomy | 108 | N |
| 1 | 1.58 | + | structural | 8.4 | 63.6 | 72 | lesionectomy | 24 | Y |
| 1 | 1.59 | + | structural | 48 | 48 | 96 | lesionectomy | 12 | N |
| 1 | 1.60 | + | structural | 27.6 | 20.4 | 48 | other resective surgery | 132 | N |
| 1 | 1.61 | + | structural | 30 | 30 | 60 | multilobar | 24 | N |
| 1 | 1.62 | + | structural | 1.8 | 46.2 | 48 | other resective surgery | 60 | Y |
| 1 | 1.63 | + | structural | 144 | 36 | 180 | other resective surgery | 24 | N |
| 1 | 1.64 | + | structural | 1.8 | 142.2 | 144 | lesionectomy | 24 | N |
| 1 | 1.65 | + | structural | 8.4 | 39.6 | 48 | lesionectomy | 120 | N |
| 1 | 1.66 | + | structural | 2.4 | 57.6 | 60 | multilobar | 24 | Y |
| 1 | 1.67 | + | structural | 6 | 114 | 120 | other resective surgery | 12 | N |
| 1 | 1.68 | + | structural | 0.12 | 1.68 | 1.8 | hemispherectomy | 48 | Y |
| 1 | 1.69 | + | structural | 24 | 36 | 60 | lesionectomy | 96 | N |
| 1 | 1.70 | + | structural | 2.4 | 21.6 | 24 | lesionectomy | 12 | Y |
| 1 | 1.71 | + | structural | 6 | 42 | 48 | lesionectomy | 60 | N |
| 1 | 1.72 | + | structural | 7.2 | 100.8 | 108 | lesionectomy | 60 | Y |
| 1 | 1.73 | - | structural | 66 | 66 | 132 | other resective surgery | 60 | N |
| 1 | 1.74 | + | structural | 8.4 | 51.6 | 60 | other resective surgery | 60 | N |
| 1 | 1.75 | + | structural | 1.2 | 70.8 | 72 | other resective surgery | 108 | Y |
| 1 | 1.76 | + | structural | 4.8 | 31.2 | 36 | multilobar | 12 | Y |
| 1 | 1.77 | + | structural | 6 | 66 | 72 | hemispherectomy | 12 | N |
| 1 | 1.78 | + | structural | 1.2 | 3.6 | 4.8 | multilobar | 168 | Y |
| 1 | 1.79 | + | structural | 0.6 | 11.4 | 12 | lesionectomy | 36 | N |
| 1 | 1.80 | - | structural | 84 | 48 | 132 | lesionectomy | 36 | N |
| 2 | 2.01 | + | structural | -- | -- | -- | lesionectomy | -- | Y |
| 2 | 2.02 | + | structural | -- | -- | -- | lesionectomy | -- | Y |
| 2 | 2.03 | + | structural | -- | -- | -- | hemispherectomy | -- | Y |
| 2 | 2.04 | + | structural | -- | -- | -- | hemispherectomy | -- | Y |
| 2 | 2.05 | + | structural | -- | -- | -- | lesionectomy | -- | Y |
| 2 | 2.06 | + | structural | -- | -- | -- | lesionectomy | -- | Y |
| 4 | 4.01 | - | genetic | 10.8 | 10.8 | 21.6 | hemispherectomy | 24 | Y |
| 4 | 4.02 | + | structural/genetic | 4.8 | 18 | 22.8 | hemispherectomy | 15 | Y |
| 4 | 4.03 | + | structural | 42 | 6 | 48 | hemispherectomy | 6 | Y |
| 4 | 4.04 | + | genetic (TSC) | 6 | 90 | 96 | frontal | 26 | N |
| 4 | 4.05 | + | genetic (TSC) | 6 | 33 | 39 | frontal | 30 | Y |
| 4 | 4.06 | + | genetic (TSC) | 36 | 84 | 120 | temporal | 23 | Y |
| 4 | 4.07 | + | structural | 3 | 6 | 9 | hemispherectomy | 24 | Y |
| 4 | 4.08 | + | structural | 7.2 | 36 | 42 | temporal | 24 | Y |
| 4 | 4.09 | + | structural | 16.8 | 8.4 | 37.2 | multilobar | 36 | Y |
| 4 | 4.10 | + | structural | 4.8 | 19.2 | 24 | hemispherectomy | 15 | Y |
| 4 | 4.11 | + | structural | 1.2 | 28.8 | 30 | hemispherectomy | 36 | Y |
| 4 | 4.12 | + | structural | 18 | 96 | 114 | temporal | 38 | Y |
| 4 | 4.13 | + | structural | 18 | 10.8 | 28.8 | hemispherectomy | 27 | Y |
| 4 | 4.14 | - | genetic (TSC) | 3.6 | 32.4 | 36 | parietal | 22 | N |
| 4 | 4.15 | + | structural | 7.2 | 48 | 55.2 | hemispherectomy | 28 | Y |
| 4 | 4.16 | - | genetic | 3.6 | 25.2 | 28.8 | hemispherectomy | 24 | Y |
| 4 | 4.17 | - | structural | 6 | 54 | 60 | hemispherectomy | 28 | N |
| 4 | 4.18 | - | structural | 1.2 | 26.4 | 27.6 | hemispherectomy | 15 | Y |
| 4 | 4.19 | + | structural | 1.2 | 8.4 | 9.6 | hemispherectomy | 36 | Y |
| 4 | 4.20 | + | structural | 1.8 | 7.8 | 9.6 | hemispherectomy | 27 | Y |
| 4 | 4.21 | + | structural | 48 | 54 | 102 | hemispherectomy | 50 | N |
| 4 | 4.22 | + | structural | 12 | 12 | 24 | hemispherectomy | 42 | Y |
| 4 | 4.23 | + | structural | 1.2 | 226.8 | 228 | hemispherectomy | 46 | N |
| 4 | 4.24 | - | structural/genetic (Down) | 6 | 3.6 | 9.6 | temporal | 20 | Y |
| 4 | 4.25 | + | genetic (TSC) | 3 | 57 | 60 | frontal | 10 | N |
| 4 | 4.26 | + | genetic (TSC) | 84 | 36 | 120 | frontal | 48 | N |
| 4 | 4.27 | + | structural | 36 | 192 | 228 | hemispherectomy | 20 | Y |
| 4 | 4.28 | + | structural | 24 | 24 | 48 | hemispherectomy | 56 | Y |
| 4 | 4.29 | + | genetic (TSC) | 6 | 36 | 42 | multilobar | 12 | N |
| 4 | 4.30 | + | genetic (TSC) | 6 | 21.6 | 27.6 | multilobar | 48 | Y |
| 4 | 4.31 | + | structural | 132 | 24 | 156 | hemispherectomy | 36 | Y |
| 4 | 4.32 | + | structural | 6 | 15.6 | 21.6 | hemispherectomy | 26 | Y |
| 4 | 4.33 | + | structural | 3 | 9 | 10.8 | multilobar | 60 | N |
| 4 | 4.34 | - | structural | 3.6 | 38.4 | 42 | multilobar | 20 | N |
| 4 | 4.35 | - | structural | 6 | 90 | 96 | hemispherectomy | 54 | N |
| 4 | 4.36 | - | structural | 21.6 | 50.4 | 72 | frontal | 9 | Y |
| 4 | 4.37 | - | structural | 4.8 | 40.2 | 45 | multilobar | 54 | N |
| 4 | 4.38 | - | structural | 3 | 12.6 | 15.6 | hemispherectomy | 58 | Y |
| 4 | 4.39 | + | structural | 54 | 6 | 60 | hemispherectomy | 15 | Y |
| 4 | 4.40 | - | structural | 4.8 | 28.2 | 33 | hemispherectomy | 67 | Y |
| 4 | 4.41 | + | genetic (TSC) | 3 | 6 | 9 | multilobar | 72 | Y |
| 4 | 4.42 | + | genetic (TSC) | 10.8 | 25.2 | 36 | other resective surgery | 68 | N |
| 4 | 4.43 | - | structural | 8.4 | 75.6 | 84 | multilobar | 76 | N |
| 4 | 4.44 | + | structural | 3.6 | 8.4 | 12 | hemispherectomy | 76 | Y |
| 4 | 4.45 | - | structural | 12 | 9.6 | 21.6 | hemispherectomy | 70 | Y |
| 4 | 4.46 | - | structural | 21.6 | 66 | 87.6 | hemispherectomy | 80 | N |
| 4 | 4.47 | + | genetic (TSC) | 12 | 132 | 144 | lesionectomy | 80 | N |
| 4 | 4.48 | - | structural | 18 | 36 | 54 | hemispherectomy | 68 | Y |
| 4 | 4.49 | + | structural | 1.8 | 0.6 | 2.4 | hemispherectomy | 9 | Y |
| 4 | 4.50 | + | structural | 84 | 84 | 168 | multilobar | 86 | Y |
| 4 | 4.51 | - | genetic | 8.4 | 29.4 | 37.8 | other resective surgery | 108 | N |
| 4 | 4.52 | + | structural | 30 | 36 | 66 | hemispherectomy | 45 | Y |
| 4 | 4.53 | + | genetic (TSC) | 1.2 | 54 | 55.2 | multilobar | 80 | Y |
| 4 | 4.54 | + | structural | 60 | 132 | 192 | hemispherectomy | 50 | Y |
| 4 | 4.55 | - | structural | 8.4 | 99.6 | 108 | hemispherectomy | 96 | N |
| 4 | 4.56 | + | genetic (TSC) | 1.2 | 19.8 | 21 | lesionectomy | 80 | Y |
| 4 | 4.57 | + | structural | 6 | 24 | 30 | hemispherectomy | 120 | Y |
| 4 | 4.58 | + | structural | 3.6 | 4.8 | 8.4 | multilobar | 24 | Y |
| 4 | 4.59 | + | genetic (TSC) | 8.4 | 117.6 | 126 | lesionectomy | 36 | Y |
| 4 | 4.60 | - | structural | 24 | 48 | 72 | multilobar | 19 | Y |
| 4 | 4.61 | + | genetic (TSC) | 3 | 93 | 96 | multilobar | 15 | Y |
| 4 | 4.62 | + | structural | 4.8 | 21 | 25.2 | hemispherectomy | 18 | Y |
| 4 | 4.63 | + | genetic (TSC) | 1.2 | 69.6 | 70.8 | frontal | 100 | N |
| 4 | 4.64 | + | genetic (TSC) | 6 | 78 | 84 | hemispherectomy | 120 | Y |
| 4 | 4.65 | + | structural | 1.8 | 39 | 40.8 | hemispherectomy | 96 | Y |
| 5 | 5.01 | + | structural | -- | -- | 8 | other resective surgery | 15 | Y |
| 5 | 5.02 | + | structural | -- | -- | 7 | other resective surgery | 45 | N |
| 5 | 5.03 | - | structural | -- | -- | 33 | other resective surgery | 47 | Y |
| 5 | 5.04 | - | structural | -- | -- | 16 | other resective surgery | 51 | Y |
| 5 | 5.05 | - | structural | -- | -- | 13 | other resective surgery | 28 | N |
| 5 | 5.06 | + | structural | -- | -- | 7 | occipital | 17 | N |
| 5 | 5.07 | - | structural | -- | -- | 33 | other resective surgery | 31 | Y |
| 5 | 5.08 | - | unknown | -- | -- | 43 | other resective surgery | 9 | Y |
| 5 | 5.09 | - | structural | -- | -- | 22 | other resective surgery | 17 | Y |
| 5 | 5.10 | - | structural | -- | -- | 9 | other resective surgery | 21 | Y |
| 5 | 5.11 | + | structural | -- | -- | 5 | other resective surgery | 34 | Y |
| 5 | 5.12 | - | structural | -- | -- | 38 | parietal | 12 | N |
| 5 | 5.13 | + | structural | -- | -- | 9 | other resective surgery | 25 | N |
| 5 | 5.14 | - | structural | -- | -- | 6 | other resective surgery | 18 | Y |
| 5 | 5.15 | - | structural | -- | -- | 44 | other resective surgery | 4 | Y |
| 5 | 5.16 | - | structural | -- | -- | 14 | other resective surgery | 62 | Y |
| 5 | 5.17 | + | structural | -- | -- | 10 | hemispherectomy | 40 | N |
| 5 | 5.18 | - | structural | -- | -- | 18 | hemispherectomy | 14 | Y |
| 5 | 5.19 | - | structural | -- | -- | 18 | hemispherectomy | 8 | Y |
| 5 | 5.20 | + | structural | -- | -- | 19 | hemispherectomy | 12 | N |
| 5 | 5.21 | + | structural | -- | -- | 17 | hemispherectomy | 29 | N |
| 5 | 5.22 | + | structural | -- | -- | 8 | hemispherectomy | 45 | Y |
| 5 | 5.23 | + | structural | -- | -- | 26 | other resective surgery | 67 | Y |
| 6 | 6.01 | + | structural | 3 | 7 | 10 | hemispherectomy | 132 | Y |
| 6 | 6.02 | + | structural | 1.5 | 10.5 | 12 | hemispherectomy | 24 | Y |
| 6 | 6.03 | + | genetic | 7 | 10 | 17 | hemispherectomy | 132 | N |
| 6 | 6.04 | + | structural | 15 | 5 | 20 | temporal | 120 | Y |
| 6 | 6.05 | + | structural | 3 | 10 | 13 | multilobar | 72 | N |
| 6 | 6.06 | + | structural | 5 | 6 | 11 | temporal | 72 | N |
| 6 | 6.07 | + | structural | 2.5 | 69.5 | 72 | hemispherectomy | 96 | N |
| 6 | 6.08 | + | structural | 5 | 19 | 24 | temporal | 72 | N |
| 6 | 6.09 | + | structural | 0 | 30 | 30 | hemispherectomy | 60 | N |
| 6 | 6.10 | + | structural | 9 | 20 | 29 | multilobar | 84 | Y |
| 6 | 6.11 | + | structural | 17 | 10 | 27 | temporal | 84 | Y |
| 6 | 6.12 | + | structural | 10 | 14 | 24 | multilobar | 12 | N |
| 6 | 6.13 | + | structural | 30 | 6 | 36 | multilobar | 12 | N |
| 6 | 6.14 | + | structural | 9 | 56 | 65 | temporal | 24 | Y |
| 6 | 6.15 | + | structural | 0 | 10 | 10 | hemispherectomy | 12 | N |
| 6 | 6.16 | + | structural | 48 | 48 | 96 | hemispherectomy | 36 | N |
| 6 | 6.17 | + | structural | 36 | 4 | 40 | temporal | 96 | Y |
| 6 | 6.18 | + | structural | 1 | 21 | 22 | multilobar | 6 | N |
| 6 | 6.19 | + | structural | 2.5 | 22.5 | 25 | multilobar | 12 | Y |
| 6 | 6.20 | + | structural | 8 | 12 | 20 | hemispherectomy | 72 | Y |
| 6 | 6.21 | + | structural | 1.5 | 2.5 | 4 | multilobar | 24 | Y |
| 6 | 6.22 | + | structural | 4 | 46 | 50 | hemispherectomy | 60 | Y |
| 6 | 6.23 | + | structural | 2 | 7 | 9 | occipital | 84 | Y |
| 6 | 6.24 | + | structural | 12 | 11 | 23 | multilobar | 60 | Y |
| 6 | 6.25 | + | structural | 4 | 25 | 29 | hemispherectomy | 36 | N |
| 6 | 6.26 | + | structural | 5 | 2 | 7 | multilobar | 48 | Y |
| 6 | 6.27 | + | structural | 8 | 27 | 35 | multilobar | 12 | Y |
| 6 | 6.28 | + | structural | 8 | 1 | 9 | frontal | 48 | Y |
| 6 | 6.29 | + | structural | 8 | 3 | 11 | temporal | 24 | Y |
| 6 | 6.30 | + | structural | 4 | 21 | 25 | multilobar | 36 | Y |
| 6 | 6.31 | + | structural | 0.5 | 8 | 9 | frontal | 6 | Y |
| 6 | 6.32 | + | structural | 8 | 14 | 22 | hemispherectomy | 24 | Y |
| 6 | 6.33 | + | structural | 4 | 54 | 58 | parietal | 24 | N |
| 6 | 6.34 | + | genetic (TSC) | 0 | 68 | 68 | multilobar | 108 | N |
| 6 | 6.35 | + | structural | 4 | 28 | 32 | frontal | 72 | N |
| 6 | 6.36 | + | structural | 24 | 19 | 43 | hemispherectomy | 108 | N |
| 6 | 6.37 | + | structural | 0 | 2 | 2 | hemispherectomy | 120 | N |
| 6 | 6.38 | + | structural | 21 | 9 | 30 | frontal | 180 | Y |
| 6 | 6.39 | + | structural | 0.25 | 3.75 | 4 | hemispherectomy | 192 | N |
| 6 | 6.40 | + | structural | 6 | 18 | 24 | hemispherectomy | 96 | Y |
| 6 | 6.41 | + | structural | 0 | 10 | 10 | hemispherectomy | 36 | N |
| 6 | 6.42 | + | structural | 8 | 20 | 28 | hemispherectomy | 108 | Y |
| 6 | 6.43 | + | structural | 0.75 | 3.25 | 4 | hemispherectomy | 84 | Y |
| 6 | 6.44 | + | structural | 0 | 6 | 6 | hemispherectomy | 48 | Y |
| 6 | 6.45 | + | genetic (TSC) | 3.5 | 56.5 | 60 | multilobar | 6 | Y |
| 6 | 6.46 | + | structural | 2 | 8 | 10 | hemispherectomy | 72 | N |
| 6 | 6.47 | + | structural | 1 | 3 | 4 | lesionectomy | 72 | N |
| 6 | 6.48 | + | structural | 0 | 4 | 4 | hemispherectomy | 12 | Y |
| 6 | 6.49 | + | structural | 4 | 10 | 14 | lesionectomy | 120 | N |
| 6 | 6.50 | + | structural | 1 | 18 | 19 | parietal | 12 | N |
| 6 | 6.51 | + | structural | 0 | 5 | 5 | hemispherectomy | 228 | N |
| 6 | 6.52 | + | structural | 0 | 17 | 17 | hemispherectomy | 24 | Y |
| 6 | 6.53 | + | structural | 2.5 | 5.5 | 8 | hemispherectomy | 24 | Y |
| 6 | 6.54 | + | structural | 8 | 18 | 26 | frontal | 18 | Y |
| 6 | 6.55 | + | structural | 3 | 5 | 8 | hemispherectomy | 6 | Y |
| 6 | 6.56 | + | structural | 2 | 44 | 46 | multilobar | 108 | N |
| 6 | 6.57 | + | Structural/genetic  (POLG) | 39 | 9 | 48 | temporal | 0 | Y |
| 6 | 6.58 | + | structural | 4 | 6 | 10 | hemispherectomy | 24 | Y |
| 6 | 6.59 | + | structural | 0 | 6 | 6 | multilobar | 0 | Y |
| 6 | 6.60 | + | structural | 9 | 10 | 19 | temporal | 108 | N |
| 6 | 6.61 | + | structural | 0 | 6 | 6 | hemispherectomy | 48 | Y |
| 6 | 6.62 | + | structural | 6 | 1 | 7 | temporal | 12 | Y |
| 6 | 6.63 | + | structural | 3 | 20 | 23 | frontal | 24 | N |
| 6 | 6.64 | + | structural | 8 | 4 | 12 | hemispherectomy | 12 | Y |
| 6 | 6.65 | + | Structural/genetic  (COLD4A1) | 5 | 7 | 12 | multilobar | 24 | N |
| 6 | 6.66 | + | structural | 9 | 9 | 18 | hemispherectomy | 7.2 | Y |
| 6 | 6.67 | + | structural | 6 | 9 | 15 | hemispherectomy | 24 | N |
| 6 | 6.68 | + | structural | 10 | 20 | 30 | temporal | 12 | Y |
| 6 | 6.69 | + | structural | 4 | 10 | 14 | frontal | 27.6 | Y |
| 6 | 6.70 | + | structural | 0 | 2 | 2 | hemispherectomy | 132 | Y |
| 7 | 7.01 | - | structural | 2 | 92 | 94 | frontal | 34 | Y |
| 7 | 7.02 | - | unknown | 3 | 56 | 59 | frontal | 39 | Y |
| 7 | 7.03 | - | structural | 7 | 25 | 32 | frontal | 36 | Y |
| 7 | 7.04 | - | structural | 20 | 19 | 39 | frontal | 35 | Y |
| 7 | 7.05 | - | structural | 2 | 40 | 42 | other resective surgery | 12 | Y |
| 7 | 7.06 | - | unknown | 8 | 15 | 23 | frontal | 36 | Y |
| 7 | 7.07 | - | structural | 20 | 22 | 42 | frontal | 35 | N |
| 7 | 7.08 | - | structural | 3 | 113 | 116 | frontal | 39 | N |
| 7 | 7.09 | - | structural | 3 | 45 | 48 | frontal | 21 | Y |
| 8 | 8.01 | - | unknown | -- | -- | -- | multilobar | 48 | N |
| 8 | 8.02 | - | unknown | -- | -- | -- | multilobar | 48 | Y |
| 8 | 8.03 | + | structural | -- | -- | -- | multilobar | 36 | Y |
| 8 | 8.04 | + | structural | -- | -- | -- | multilobar | 24 | Y |
| 8 | 8.05 | + | structural | -- | -- | -- | multilobar | 48 | Y |
| 8 | 8.06 | + | structural | -- | -- | -- | other resective surgery | 24 | Y |
| 8 | 8.07 | + | structural | -- | -- | -- | multilobar | 36 | Y |
| 8 | 8.08 | - | unknown | -- | -- | -- | multilobar | 36 | N |
| 8 | 8.09 | + | structural | -- | -- | -- | multilobar | 24 | Y |
| 8 | 8.10 | + | structural | -- | -- | -- | multilobar | 24 | N |
| 8 | 8.11 | + | structural | -- | -- | -- | other resective surgery | 36 | N |
| 9 | 9.01 | + | #N/A | 8 | 18 | 26 | other resective surgery | 24 | Y |
| 9 | 9.02 | + | #N/A | 1 | 24 | 25 | lesionectomy | 24 | Y |
| 9 | 9.03 | + | #N/A | 2 | 8 | 10 | hemispherectomy | 24 | Y |
| 9 | 9.04 | + | #N/A | 4 | 8 | 12 | other resective surgery | 24 | N |
| 9 | 9.05 | + | #N/A | 10 | 9 | 19 | multilobar | 24 | N |
| 9 | 9.06 | + | #N/A | 3 | 8 | 11 | hemispherectomy | 24 | Y |
| 10 | 10.01 | + | structural | 1 | 3 | 4 | other resective surgery | -- | Y |
| 10 | 10.02 | + | structural | 3 | 2 | 5 | other resective surgery | -- | Y |
| 10 | 10.03 | + | structural | 3 | 8 | 11 | other resective surgery | -- | Y |
| 10 | 10.04 | + | structural | 3 | 15 | 18 | other resective surgery | -- | Y |
| 10 | 10.05 | + | structural | 2 | 17 | 19 | other resective surgery | -- | N |
| 10 | 10.06 | + | structural | 3 | 20 | 23 | other resective surgery | -- | Y |
| 10 | 10.07 | + | structural | 5 | 23 | 28 | other resective surgery | -- | N |
| 10 | 10.08 | + | structural | 2 | 31 | 33 | other resective surgery | -- | Y |
| 10 | 10.09 | - | structural | 1 | 34 | 35 | occipital | -- | N |
| 10 | 10.10 | + | structural | 5 | 65 | 70 | other resective surgery | -- | Y |
| 10 | 10.11 | + | structural | 3 | 118 | 121 | other resective surgery | -- | Y |
| 13 | 13.01 | + | genetic (TSC) | 3.6 | 22.8 | 26.4 | hemispherectomy | 45.6 | Y |
| 13 | 13.02 | + | genetic (TSC) | 6 | 36 | 42 | lesionectomy | 14.4 | Y |
| 13 | 13.03 | + | genetic (TSC) | 4.8 | 13.2 | 18 | lesionectomy | 42 | Y |
| 13 | 13.04 | + | genetic (TSC) | 8.4 | 25.2 | 33.6 | hemispherectomy | 72 | Y |
| 13 | 13.05 | + | genetic (TSC) | 10.8 | 21.6 | 32.4 | hemispherectomy | 26.4 | N |
| 13 | 13.06 | + | genetic (TSC) | 14.4 | 22.8 | 37.2 | hemispherectomy | 61.2 | Y |
| 13 | 13.07 | + | genetic (TSC) | 7.2 | 12 | 19.2 | lesionectomy | 36 | N |
| 13 | 13.08 | + | genetic (TSC) | 4.8 | 16.8 | 21.6 | lesionectomy | 24 | N |
| 13 | 13.09 | + | genetic (TSC) | 9.6 | 49.2 | 58.8 | hemispherectomy | 19.2 | Y |
| 13 | 13.10 | + | genetic (TSC) | 10.8 | 43.2 | 54 | lesionectomy | 28.8 | Y |
| 13 | 13.11 | + | genetic (TSC) | 13.2 | 37.2 | 50.4 | hemispherectomy | 32.4 | Y |
| 13 | 13.12 | + | genetic (TSC) | 3.6 | 92.4 | 96 | hemispherectomy | 30 | N |
| 13 | 13.13 | + | genetic (TSC) | 28.8 | 48 | 76.8 | hemispherectomy | 38.4 | Y |
| 13 | 13.14 | + | genetic (TSC) | 6 | 60 | 66 | lesionectomy | 54 | Y |
| 13 | 13.15 | + | genetic (TSC) | 38.4 | 39.6 | 78 | hemispherectomy | 24 | N |
| 13 | 13.16 | + | genetic (TSC) | 8.4 | 58.8 | 67.2 | lesionectomy | 28.8 | Y |
| 13 | 13.17 | + | genetic (TSC) | 12 | 73.2 | 85.2 | hemispherectomy | 33.6 | N |
| 14 | 14.01 | + | structural | 8 | 35 | 43 | temporal | 48 | Y |
| 14 | 14.02 | + | structural | 2 | 13 | 15 | temporal | 36 | Y |
| 14 | 14.03 | + | structural | 48 | 5 | 53 | temporal | 60 | Y |
| 14 | 14.04 | + | structural | 8 | 7 | 15 | temporal | 24 | N |
| 14 | 14.05 | + | structural | 7 | 2 | 9 | temporal | 60 | Y |
| 14 | 14.06 | + | structural | 10 | 8 | 18 | temporal | 72 | Y |
| 14 | 14.07 | + | structural | 6 | 4 | 10 | temporal | 60 | Y |
| 14 | 14.08 | - | structural | 1 | 5 | 6 | temporal | 24 | N |
| 14 | 14.09 | - | structural | 1 | 10 | 11 | temporal | 12 | N |
| 14 | 14.10 | + | structural | 3 | 3 | 6 | temporal | 12 | Y |
| 14 | 14.11 | + | structural | 12 | 31 | 43 | temporal | 72 | N |
| 14 | 14.12 | + | structural | 4 | 11 | 15 | temporal | 72 | Y |
| 14 | 14.13 | + | structural | 42 | 8 | 50 | temporal | 48 | Y |
| 14 | 14.14 | + | structural | 19 | 7 | 26 | temporal | 48 | Y |
| 14 | 14.15 | + | structural | 30 | 20 | 50 | temporal | 24 | N |
| 14 | 14.16 | + | structural | 1 | 7 | 8 | temporal | 276 | N |
| 14 | 14.17 | + | structural/genetic (NF1) | 2 | 43 | 45 | temporal | 24 | N |
| 14 | 14.18 | + | senetic (TSC) | 9 | 41 | 50 | temporal | 72 | Y |
| 14 | 14.19 | + | structural | 14 | 19 | 33 | temporal | 168 | Y |
| 14 | 14.20 | + | structural | 8 | 13 | 21 | temporal | 120 | Y |
| 15 | 15.01 | + | structural | 18 | 18 | 36 | other resective surgery | 24 | Y |
| 15 | 15.02 | + | structural | 18 | 90 | 108 | other resective surgery | 24 | Y |
| 15 | 15.03 | + | structural | 18 | 114 | 132 | other resective surgery | 84 | N |
| 15 | 15.04 | - | unknown | 20 | 196 | 216 | other resective surgery | 24 | N |
| 15 | 15.05 | + | structural | 23 | 25 | 48 | other resective surgery | 24 | Y |
| 15 | 15.06 | + | structural | 30 | 138 | 168 | multilobar | 12 | Y |
| 16 | 16.01 | + | unknown | -- | -- | 2.5 | multilobar | 25 | Y |
| 16 | 16.02 | + | unknown | -- | -- | 5 | multilobar | 34 | Y |
| 16 | 16.03 | + | unknown | -- | -- | 20.4 | multilobar | 12 | Y |
| 16 | 16.04 | + | unknown | -- | -- | 25.2 | multilobar | 69 | Y |
| 16 | 16.05 | + | genetic | -- | -- | 80.4 | multilobar | 21 | N |
| 16 | 16.06 | + | unknown | -- | -- | 188.4 | multilobar | 65 | N |
| 16 | 16.07 | + | unknown | -- | -- | 36 | multilobar | 41 | Y |
| 16 | 16.08 | + | unknown | -- | -- | 54 | multilobar | 27 | N |
| 16 | 16.09 | + | unknown | -- | -- | 58.8 | multilobar | 88 | Y |
| 16 | 16.10 | + | unknown | -- | -- | 195.6 | multilobar | 86 | N |
| 17 | 17.01 | + | structural | 1 | 6.4 | 7.4 | lesionectomy | -- | N |
| 17 | 17.02 | + | structural | 4 | 13.2 | 17.2 | hemispherectomy | -- | N |
| 17 | 17.03 | + | structural | 10.5 | 14.4 | 24.9 | frontal | -- | Y |
| 17 | 17.04 | + | structural | 8 | 26.8 | 34.8 | hemispherectomy | -- | Y |
| 17 | 17.05 | + | structural | 33 | 4.6 | 37.6 | lesionectomy | -- | Y |
| 17 | 17.06 | + | structural | 30 | 14.5 | 44.5 | temporal | -- | Y |
| 17 | 17.07 | - | unknown | 1.7 | 54.8 | 56.5 | other resective surgery | -- | N |
| 17 | 17.08 | + | structural | 36 | 24.7 | 60.7 | hemispherectomy | -- | N |
| 17 | 17.09 | + | structural | 11 | 98.3 | 109.3 | lesionectomy | -- | N |
| 17 | 17.10 | + | structural | 11 | 6.4 | 17.4 | temporal | -- | N |
| 17 | 17.11 | + | structural | 1 | 26.1 | 27.1 | multilobar | -- | Y |
| 18 | 18.01 | + | structural | 2 | 6 | 8 | parietal | 4 | Y |
| 18 | 18.02 | + | structural | 0 | 5 | 5 | frontal | 110 | Y |
| 18 | 18.03 | + | structural | 2.5 | 17.5 | 20 | occipital | 13 | Y |
| 18 | 18.04 | + | structural | 7 | 28 | 35 | frontal | 83 | Y |
| 18 | 18.05 | + | structural | 3 | 25 | 28 | frontal | 65 | Y |
| 18 | 18.06 | + | structural | 3 | 62 | 65 | frontal | 48 | Y |
| 18 | 18.07 | + | structural | 20 | 19 | 39 | parietal | 28 | Y |
| 18 | 18.08 | + | structural | 3 | 9 | 12 | frontal | 32 | N |
| 18 | 18.09 | + | structural | 7 | 33 | 40 | parietal | 9 | Y |
| 18 | 18.10 | + | structural | 3 | 11 | 14 | parietal | 20 | N |
| 18 | 18.11 | + | structural | 1 | 32 | 33 | frontal | 13 | N |
| 19 | 19.01 | - | structural | 84 | 120 | 204 | frontal | 85 | N |
| 19 | 19.02 | + | genetic (TSC) | 12 | 60 | 72 | lesionectomy | 72 | N |
| 19 | 19.03 | + | structural | 72 | 24 | 96 | lesionectomy | 59 | N |
| 19 | 19.04 | + | structural | 72 | 12 | 84 | lesionectomy | 57 | Y |
| 19 | 19.05 | + | structural | 36 | 96 | 132 | multilobar | 51 | N |
| 19 | 19.06 | + | structural | 156 | 108 | 264 | multilobar | 47 | Y |
| 19 | 19.07 | + | structural | 10.8 | 37.2 | 48 | lesionectomy | 40 | N |
| 19 | 19.08 | + | structural | 21.6 | 38.4 | 60 | lesionectomy | 40 | N |
| 19 | 19.09 | - | structural | 27.6 | 44.4 | 72 | multilobar | 38 | N |
| 19 | 19.10 | - | structural | 54 | 66 | 120 | frontal | 38 | Y |
| 19 | 19.11 | + | structural | 60 | 12 | 72 | lesionectomy | 38 | Y |
| 19 | 19.12 | + | structural | 22.8 | 13.2 | 36 | hemispherectomy | 38 | N |
| 19 | 19.13 | + | genetic (TSC) | 99.6 | 8.4 | 108 | lesionectomy | 37 | Y |
| 19 | 19.14 | + | structural | 6 | 78 | 84 | lesionectomy | 36 | N |
| 19 | 19.15 | + | structural | 6 | 30 | 36 | hemispherectomy | 33 | N |
| 19 | 19.16 | + | structural | 12 | 180 | 192 | lesionectomy | 30 | Y |
| 19 | 19.17 | - | structural | 7.2 | 40.8 | 48 | multilobar | 25 | N |
| 19 | 19.18 | - | structural | 72 | 36 | 108 | multilobar | 25 | N |
| 19 | 19.19 | + | structural | 78 | 18 | 96 | lesionectomy | 24 | N |
| 19 | 19.20 | + | genetic (TSC) | 15.6 | 80.4 | 96 | lesionectomy | 23 | Y |
| 19 | 19.21 | + | structural | 102 | 18 | 120 | lesionectomy | 22 | Y |
| 19 | 19.22 | + | structural | 48 | 72 | 120 | lesionectomy | 23 | Y |
| 19 | 19.23 | + | structural | 60 | 48 | 108 | lesionectomy | 19 | N |
| 19 | 19.24 | + | structural | 55.2 | 4.8 | 60 | multilobar | 18 | N |
| 19 | 19.25 | + | structural | 48 | 72 | 120 | lesionectomy | 17 | Y |
| 19 | 19.26 | + | structural | 72 | 36 | 108 | hemispherectomy | 16 | Y |
| 20 | 20.01 | + | genetic (TSC) | 60 | 84 | 144 | multilobar | 36 | Y |
| 20 | 20.02 | + | structural | 12 | 84 | 96 | multilobar | 24 | N |
| 20 | 20.03 | + | structural | 24 | 24 | 48 | hemispherectomy | 24 | Y |
| 20 | 20.04 | + | structural | 84 | 96 | 180 | multilobar | 24 | Y |
| 20 | 20.05 | + | structural | 48 | 60 | 108 | multilobar | 18 | Y |
| 20 | 20.06 | + | structural | 36 | 132 | 168 | multilobar | 12 | N |
| 20 | 20.07 | + | structural | 24 | 324 | 348 | hemispherectomy | 18 | Y |
| 20 | 20.08 | + | structural | 30 | 30 | 60 | multilobar | 12 | Y |
| 20 | 20.09 | + | structural | 6 | 30 | 36 | multilobar | 12 | Y |
| 20 | 20.10 | + | structural | 60 | 96 | 156 | multilobar | 18 | Y |
| 20 | 20.11 | + | structural | 96 | 60 | 156 | multilobar | 12 | Y |
| 20 | 20.12 | + | structural | 96 | 312 | 408 | multilobar | 12 | N |
| 20 | 20.13 | + | structural | 12 | 36 | 48 | multilobar | 12 | Y |
| 20 | 20.14 | + | structural | 24 | 36 | 60 | multilobar | 12 | N |
| 20 | 20.15 | + | structural | 30 | 18 | 48 | multilobar | 24 | N |
| 20 | 20.16 | + | structural | 18 | 18 | 36 | multilobar | 48 | Y |
| 20 | 20.17 | + | structural | 36 | 84 | 120 | multilobar | 12 | N |
| 20 | 20.18 | + | structural | 24 | 48 | 72 | lesionectomy | 18 | Y |
| 20 | 20.19 | + | structural | 6 | 78 | 84 | multilobar | 18 | Y |
| 21 | 21.01 | + | structural | 0 | 9 | 9 | hemispherectomy | -- | N |
| 21 | 21.02 | + | structural | 7 | 8 | 15 | hemispherectomy | -- | Y |
| 21 | 21.03 | + | structural | 7 | 16 | 23 | lesionectomy | -- | Y |
| 21 | 21.04 | + | structural | 11 | 6 | 17 | temporal | -- | Y |
| 21 | 21.05 | + | structural | 2 | 27 | 29 | frontal | -- | Y |
